# Clarifying the relationship between mental illness and recidivism using machine learning: A retrospective study

**DOI:** 10.1371/journal.pone.0297448

**Published:** 2024-02-23

**Authors:** Talia R. Cohen, Gaylen E. Fronk, Kent A. Kiehl, John J. Curtin, Michael Koenigs

**Affiliations:** 1 Department of Psychology, University of Wisconsin-Madison, Madison, Wisconsin, United States of America; 2 Department of Psychiatry, University of Wisconsin-Madison, Madison, Wisconsin, United States of America; 3 Departments of Psychology, The Mind Research Network, Neuroscience and Law, University of New Mexico, Albuquerque, New Mexico, United States of America; Universidad de Costa Rica, COSTA RICA

## Abstract

**Objective:**

There is currently inconclusive evidence regarding the relationship between recidivism and mental illness. This retrospective study aimed to use rigorous machine learning methods to understand the unique predictive utility of mental illness for recidivism in a general population (i.e.; not only those with mental illness) prison sample in the United States.

**Method:**

Participants were adult men (n = 322) and women (n = 72) who were recruited from three prisons in the Midwest region of the United States. Three model comparisons using Bayesian correlated t-tests were conducted to understand the incremental predictive utility of mental illness, substance use, and crime and demographic variables for recidivism prediction. Three classification statistical algorithms were considered while evaluating model configurations for the t-tests: elastic net logistic regression (GLMnet), k-nearest neighbors (KNN), and random forests (RF).

**Results:**

Rates of substance use disorders were particularly high in our sample (86.29%). Mental illness variables and substance use variables did not add predictive utility for recidivism prediction over and above crime and demographic variables. Exploratory analyses comparing the crime and demographic, substance use, and mental illness feature sets to null models found that only the crime and demographics model had an increased likelihood of improving recidivism prediction accuracy.

**Conclusions:**

Despite not finding a direct relationship between mental illness and recidivism, treatment of mental illness in incarcerated populations is still essential due to the high rates of mental illnesses, the legal imperative, the possibility of decreasing institutional disciplinary burden, the opportunity to increase the effectiveness of rehabilitation programs in prison, and the potential to improve meaningful outcomes beyond recidivism following release.

## Introduction

The United States has the most incarcerated people per capita of any nation in the world [1]. Two issues that plague the United States criminal justice system are high recidivism rates and a disproportionate number of individuals with mental health problems. There is currently inconclusive evidence regarding the relationship between recidivism and mental illness. It is imperative to understand this relationship in order to inform policy and interventions to decrease criminal justice system involvement for individuals with mental illness.

The prevalence rates for most mental illnesses are higher in U.S. prisons than in the general population [2]. Two million people with mental illness are incarcerated in jails alone each year [3]. A study by the Bureau of Justice Statistics that surveyed close to 15,000 people in prison and 7,000 people in jail across the United States reported that 56% of individuals in prison and 65% in jail had a mental health problem [4]. The magnitude of the mental health treatment need in carceral settings far exceeds the ability of the criminal legal system to provide adequate treatment.

Recidivism is pervasive among individuals who have been incarcerated. Of the more than 600,000 people are released from state and federal prisons in the United States each year [5]. 44% recidivated within the first year of release and over 80% re-offend within 10 years of release [6] Due to the large numbers of individuals returning to prison, it is critical to understand the factors that contribute to the cycle of crime.

Interest in risk factors for recidivism dates back to the early 20^th^ century [7]. Much research on recidivism has looked at explanatory models, attempting to uncover the variables that explain the most variance in recidivism outcomes. In this explanatory approach, two meta-analyses have reviewed the literature on the most significant correlates of adult recidivism. Gendreau et al. (1996) included 131 studies with 1141 correlations and found that the strongest associations with general recidivism were criminal history, antisocial personality, and criminogenic needs. The results of the meta-analysis indicated that psychiatric symptomatology did not correlate with recidivism, though the finding was based on only a few effect sizes. Much of the early work on predicting outcomes was based on data from Canada, which has very different social, demographic and criminal justice processes than the United States. A more recent follow-up meta-analysis included correlations with adult recidivism from 19 studies in just the United States. In contrast to the results from Gendreau et al. (1996), Katsiyannis et al found problematic substance use followed by other mental health issues had the strongest associations with general recidivism [8].

The divergent results in these two meta-analyses concerning the relevance of mental illness for recidivism is representative of the larger discrepancy in the literature. Some research has found no link between mental illness (other than substance use disorders) and recidivism [9–11]. For example, a study of incarcerated individuals with severe mental illness (SMI), defined as individuals with psychosis or mood disorders, leaving prison found that psychiatric history did not predict re-arrest [10]. On the other hand, multiple studies have reported an association between mental illness and recidivism or reincarceration [12–17]. The presence of psychotic symptoms has been linked to an increased risk of violent recidivism [13], and a diagnosis of posttraumatic stress disorder (PTSD) was found to increase the likelihood of a new arrest [16]. People on parole with mental illness in California were almost twice as likely to recidivate within a year than those on parole without mental illness [15], and people on parole with SMI in Utah returned to prison more frequently and almost a full year sooner than those without mental illness [12]. Individuals on parole with mental illness are also more likely to have served a prior prison term than those without mental illness [17].

While the literature differs on whether mental illness diagnoses other than substance use disorders predict recidivism, the link between substance use and recidivism is more consistent [8,17,18]. A meta-analysis of general and violent recidivism in individuals with mental illness found that two of the strongest predictors of general recidivism in this population were substance use and antisocial personality disorder. Other mental health variables were not significant predictors [11]. In a sample of more than 14,000 individuals released to parole in Tennessee, a mental illness diagnosis increased the likelihood of recidivism by 14%. However, the majority of this relationship was accounted for by substance use disorders [17]. In a sample of Swiss individuals with mental illness, a substance use disorder was the only mental illness variable associated with recidivism [19]. Conversely, Sadeh & McNiel (2015) found that a PTSD diagnosis was associated with recidivism above and beyond a substance use disorder.

Given the large number of variables that are involved in recidivism prediction, it is unsurprising that machine learning, an analytic method that can handle high-dimensional predictor sets, has begun to be applied to this area (e.g., [20–26]. Many of the variables involved in recidivism prediction each account for only a small proportion of the variance in recidivism outcomes. Machine learning enables the researcher to include all variables in a single model in order to increase prediction accuracy. Previous studies investigating the relationship between mental illness and recidivism have utilized only a few predictors, typically grouped diagnoses, to operationalize the wide array of mental health symptoms [16,17,27]. Machine learning, on the other hand, provides the opportunity to include a more comprehensive assessment of mental illness.

Machine learning has multiple additional advantages when compared to the general linear model. Most prior research on the relationship between mental illness and recidivism has used statistical algorithms that assume the relationship is linear [12,13,15–17]. Due to the complexity of the factors that contribute to recidivism, it is possible that the relationship between mental illness and recidivism is nonlinear. Machine learning uses statistical algorithms that easily accommodate nonlinear relationships, as well as unanticipated interactions.

In addition, machine learning uses resampling methods like cross-validation to evaluate model performance in new data, rather than in the same data that were used to estimate the parameters as is the case with the general linear model. This method increases confidence in the ability to replicate findings in new samples. Cross-validation also reduces overfitting by averaging performance estimates across resampled folds, instead of relying on a single estimate to quantify the relationship between variables.

Only two studies have employed machine learning to understand the relationship between recidivism and mental illness [19,28]. Both studies were conducted in Switzerland and included only individuals with severe mental illness. Pflueger et al. (2015) used a (nonlinear) random forest algorithm to determine the predictive value of mental illness for recidivism in a sample of individuals with mental illness in Basel, Switzerland. Their sample included 379 individuals with mental illness, 269 of whom were under inpatient treatment order. Results showed that aside from substance use disorder, no other mental illness variables were significantly predictive of recidivism. Kirchebner et al. (2020) used various machine learning algorithms to identify the most important predictor variables of recidivism in their sample of 344 individuals with schizophrenia. In addition to criminal variables, anxiety upon discharge, opioid use, and time since last psychiatric inpatient treatment were identified as the most influential variables predicting recidivism.

The current project is thus the first study to use machine learning to determine the degree to which mental illness variables and substance use variables predict recidivism in a general population prison sample in the United States. The goal of this retrospective study was not to develop a machine learning model that assesses for recidivism risk. Rather, we used rigorous machine learning methods to answer an explanatory question that has been disputed in the literature about the predictive utility of mental illness for recidivism. We conducted three model comparisons to answer this question. The first comparison examined the unique, incremental predictive utility of mental illness variables and substance use variables controlling for crime and demographic variables. The second comparison tested the unique value of mental illness variables, controlling for crime, demographics, and substance use variables. And the third comparison controlled for crime, demographics, and mental illness variables and examined the unique predictive utility of substance use. This approach allowed us to parse the predictive utility of all mental illness variables (above crime and demographic variables) as well as the unique benefit of mental illness variables other than substance use disorders, providing clarification to this disputed topic in the literature.

## Methods

### Research transparency

This project was inappropriate for preregistration because components of our analysis plan evolved as we improved understanding of these analytic methods that remain somewhat novel to our research group. However, we value the principles of research transparency that are fundamental to the robustness and replicability of science and took several steps to follow emerging open science guidelines [29]. Thus, we still aimed to maximize our transparency via several complementary methods.

First, some decisions were already and irreversibly made such as the available participant pool from which the current sample is drawn and the collected predictor variables. Second, we fully describe all measures used as predictor variables, exclusionary criteria for our analysis sample, and analysis procedures within this paper. We note when we made decisions that involved researcher degrees of freedom and outline why we selected that option. Critically, our most important analytic decision–to use the Bayesian correlated t-test–was selected at project outset during a master’s thesis proposal and never changed. Many researcher degrees of freedom were restricted via cross-validation procedures, which can robustly guide analytic decisions. Cross-validation prioritizes out-of-sample prediction such that any internal analytic decisions that improve model fit in the held-in sample are only valuable to the extent that they improve prediction in the held-out sample. Finally, we completed a transparency checklist [30], which appears in S1 Text.

### Participants

Participants for the current study were adult men (*n* = 322) and women (*n =* 72) who were recruited from three prisons in the Midwest region of the United States as part of an ongoing, long-term research project with incarcerated individuals. Participants were included in the parent research project if they met the following criteria: between the ages of 18 and 55, no history of a psychotic disorder or bipolar disorder, a fourth grade reading level or above, and a score of at least 70 on the Wechsler Adult Intelligence Scale-Revised [31]. The first and last authors had access to identifiable data, which were stored separately.

Our sample for the current study included all individuals from the parent research project who provided SCID-5 data, the key data source for mental illness variables at the center of this project’s research questions. This criterion resulted in a final sample for the present study of 322 individuals incarcerated at a men’s facility and 72 individuals incarcerated at a women’s facility (total sample: 394 incarcerated adults). In accordance with DSM-5 updates, we shifted from using the SCID-IV to the SCID-5 in 2017; thus, our sample was recruited between the years of 2017–2020. Individuals with a history of psychosis and bipolar disorder were excluded from the parent study due to concerns for safety and competency to consent to research.

### Procedure

All procedures were approved by the [location masked] Institutional Review Board. Informed consent was obtained from all participants before data collection, and each participant was told that their participation in research was completely voluntary and had no impact on their incarceration status. Participants were compensated $5/hour for participation, which represents a higher wage than individuals typically receive for other employment during their incarceration but not so high as to add undue influence to participate in our study.

Participants completed two interview sessions to assess psychiatric symptoms and psychopathy. In addition, they filled out a packet of self-report questionnaires designed to address a variety of research questions associated with the ongoing parent research project; measures relevant to the present study are described below.

### Predictor variables

A full list of predictor variables is available in Table 1. Features are divided into four sets: crime, demographics, substance, and mental illness. For analyses, the crime and demographic feature sets were paired together.

**Table 1:** Feature set variables.

Feature Set	Variables	Type of variable
Demographics	Age	Continuous
	Education	Ordinal
	Ethnicity	Categorical
	Father’s education	Ordinal
	GED	Dichotomous (yes/no)
	Gender	Categorical
	Mother’s education	Ordinal
	Race	Categorical
Crime	15 types of charges	Dichotomous (yes/no)
	Charges/arrest under age 18	Dichotomous (yes/no)
Mental illness	28 diagnoses	Dichotomous (yes/no)
	20 symptom counts*	Continuous
Substance use	8 diagnoses**	Dichotomous (yes/no)
	8 symptom counts	Continuous

* The SCID-5 does not contain separate modules for current and lifetime diagnoses for certain disorders, therefore there is only one symptom count variable for some disorders.

** All substance use diagnoses were lifetime.

#### Crime variables

Crime variables were obtained from a public Department of Corrections (DOC) database as well as from the Psychopathy Checklist-Revised (PCL-R) [32] interview. For the 15 types of offense variables, a trained undergraduate coded what type of offense(s) each participant was incarcerated for at the time of their interview for the study. The charges under age 18 variable was obtained from the PCL-R interview.

#### Demographic variables

Demographic variables were collected during the initial screening process for the parent research project and included age, education, ethnicity, father’s education, GED, mother’s education, and race.

#### Mental illness variables

All participants completed the Structured Clinical Interview for the *Diagnostic and Statistical Manual of Mental Disorders* (SCID-5) [33,34]. Twenty-eight diagnoses and symptoms counts for each diagnosis were obtained from the SCID-5. We used the mood disorders, substance use disorders, anxiety disorders, obsessive–compulsive and related disorders, trauma and stressor-related disorders, and personality disorders modules. For some diagnoses, such as Panic Disorder, symptom counts were not differentiated between current and lifetime diagnosis because there are not separate current and lifetime modules. Therefore, there are current diagnosis and lifetime diagnosis variables for panic disorder, but only one symptom count. Symptom counts provide a measure of illness or sub-threshold illness severity.

#### Substance use variables

Eight substance use disorder diagnoses were obtained from the SCID-5 interviews. Only lifetime diagnoses were used for substance use, and symptom counts were obtained for each diagnosis.

### Outcome measure

Our outcome measure (recidivism) is defined retrospectively as history of a previous adult incarceration before the incarceration sentence at the time of the assessments in line with other research that has defined recidivism similarly [28,35]. History of a previous incarceration was found through an online public DOC database. Thus, individuals with a history of previous adult incarceration were classified as positive for our outcome (cases), and individuals without a history of previous adult incarceration (i.e., for whom the current incarceration at the time of assessment was their first) were classified as negative for our outcome (controls). In the current sample, 31.7% of individuals were classified as positive for recidivism.

### Analysis strategy

We used the tidymodels framework in R for our analyses. Tidymodels is a collection of multiple packages used for machine learning within the context of the tidyverse [36], another collection of packages used for data science.

#### Candidate statistical algorithms

We considered three classification statistical algorithms while evaluating model configurations: elastic net logistic regression (GLMnet), k-nearest neighbors (KNN), and random forests (RF). These algorithms differ across many characteristics (e.g., parametric vs. non-parametric, linear vs. non-linear, flexibility, complexity). Considering this variety of algorithms increases our chances of capturing the true shape of the relationship among the predictors and outcome. All of these algorithms are well-established with documented good “out of box” performance [37].

*GLMnet algorithm*. GLMnet is a regularized version of logistic regression that penalizes model complexity using a blend of L1 (lasso regression) and L2 (ridge regression) penalties to shrink (or set to zero) parameter estimates for predictors. Ultimately, only predictors that contribute meaningfully to the model (i.e., whose predictive value offsets the “penalty” of adding a predictor to the model) are retained with meaningful parameter estimates. We trained candidate GLMNet model configurations that differed across sensible values for the hyperparameters penalty (lambda) and mixture (alpha).

*K-nearest neighbors (KNN) algorithm*. KNN is a highly flexible algorithm that allows for nonlinear relationships (including interactions) and decision boundaries. An observation is classified into an outcome class based on the outcome classes of the *k* closest observations. We trained candidate KNN model configurations that differed across sensible values for the hyperparameter *k*.

*Random forest (RF) algorithm*. RF can also accommodate both nonlinear and interactive effects of predictors. Through an ensemble of classification and regression trees (CART), a smaller number of randomly chosen variables are used to create binary decision trees. We trained candidate RF model configurations that differed across sensible values for the hyperparameters mtry and minimum n. All configurations used 900 trees to account for 10-times the number of variables.

#### Feature engineering

Feature engineering is the process of creating meaningful numeric and categorical representations (i.e., features) from raw data to improve model performance. Feature engineering can include approaches that represent categorical predictors numerically (e.g., ordinal scoring, dummy coding), and address issues associated with distributional shape (e.g., Yeo-Johnson transformations for normality). Missing data can be handled via feature engineering; we used median imputation for continuous features and mode imputation for categorical features, for features with < 25% missingness (no variables had > 25% missingness). Feature engineering was conducted for each feature set separately to build the best model for that particular feature set.

#### Resampling approach

Model configurations were evaluated using 10 repeats of 10-fold cross validation. In short, 10*-*fold cross validation splits the data into 10 folds (or groups). The model is fit or trained on 9 folds and then evaluated on the final held-out fold; this process is iterated through 10 times until each fold has been used once as the held-out fold. The performance estimates from each iteration are averaged for a more stable evaluation of model performance in data that were not used to train the model. These estimates are made more stable by conducting 10 repeats of the 10-fold cross-validation (10 x 10-fold cross-validation), where 10-fold cross-validation is repeated ten times with ten unique splits of the data. The model configuration, defined as the combination of statistical algorithm and hyperparameter values, with the best performance averaged across held-out folds was selected as the best model configuration. The best selected model for each predetermined combination of feature sets was used for model comparisons (see below).

#### Performance metric

Accuracy, or the proportion of correctly classified cases, was chosen as the performance metric for our study. Accuracy is considered relatively interpretable compared to other performance metrics and enabled us to answer our question about the relative increase in predictive accuracy of mental illness variables. We were less concerned about the types of errors the models made (e.g., false positives vs. false negatives), so a metric that describes overall performance rather than specific errors was well-suited for our purposes.

#### Model comparisons

To determine the degree to which the mental illness feature set and substance use feature set predicted recidivism, we conducted three model comparisons [38]. Each model comparison compared two model configurations that differed with respect to the feature sets included. Each augmented model included one or more additional sets of features not included in the corresponding compact model, which allowed us to evaluate the unique predictive value of the differing (i.e., focal) feature set(s). Specifically, we compared models with and without mental illness + substance use feature sets, models with and without the mental illness feature set, and models with and without the substance use feature set (Table 2).

**Table 2:** Model comparisons.

Focal Feature Set(s)	Compact	Augmented
Mental illness + Substance use	Crime, Demographics	Crime, Demographics, Substance use, Mental illness
Mental illness	Crime, Demographics, Substance use	Crime, Demographics, Substance use, Mental illness
Substance use	Crime, Demographics, Mental illness	Crime, Demographics, Substance use, Mental illness

*Note*. Crime, Demographics, Substance use, and Mental illness are feature sets. Variables included in each feature set are reported in Table 1.

Our 10x10-fold cross validation procedure yielded 100 estimates of performance for each compact and augmented model. We compared these performance estimates from compact and augmented models using a Bayesian correlated t-test [38]. The Bayesian correlated t-test calculates the posterior probability (the likelihood given our data) that the augmented model performs better than the compact model for each comparison. In contrast to a frequentist approach (null hypothesis significance testing), Bayesian approaches provide the posterior probabilities of alternative hypotheses to allow us to estimate the certainty of our results. The Bayesian correlated t-test is specifically designed to adjust for non-independence in the data due to overlapping datasets used during resampling. We display the likelihood that the augmented models performed better than the compact model by plotting the posterior probability distributions for the parameter estimates of interest (i.e., differences in model performance).

### Shapley additive explanations for relative feature importance

We computed Shapley Values [39]to provide a consistent, objective explanation of feature importance (i.e., contribution to predictions) across our augmented and compact models. Within each model, Shapley values show relative feature importance of the included features. We calculated Shapley values from the 100 held-out folds (10 repeats of 10-fold cross-validation) using the DALEX package in R. We averaged the 10 Shapley values for each participant for each feature across the 10 repeats to increase stability. To calculate global importance for categories of features (e.g., cannabis use disorder diagnosis *and* symptom counts), we added Shapley values across all features in a category separately for each participant, then averaged the absolute value of the Shapley values of all features in the category across all participants.

## Results

### Sample characteristics

Demographic characteristics for our sample, grouped by recidivism status, are reported in Table 3. Gender identity data were not available for this sample; consequently, gender is assumed by the gender of the facility. One hundred and twenty-five (31.72%) participants had a previous adult incarceration, which is consistent with the three-year prison recidivism rate [40]. Thus, the baseline performance for our models was 68.3%. Compared to the state’s prison population, Black individuals (27.9%) are underrepresented in our sample [41].

**Table 3:** Demographic characteristics by recidivism status.

Variable			% (*N*) or *M (SD)*			
		No Recidivism		Recidivism	Total	
Age		33.2 (8.35)		37.3 (8.06)	34.5 (8.47)	
Gender						
Male		79.55% (214)		86.4% (108)	81.7% (322)	
Female		20.45% (55)		13.6% (17)	18.3% (72)	
Race						
Asian		1.21% (3)		.80% (1)	1.02% (4)	
Black		25.28% (68)		33.60% (42)	27.90% (110)	
Multiracial		7.06% (19)		3.20% (4)	5.84% (23)	
Native American		5.20% (14)		3.20% (4)	4.57% (18)	
White		61.34% (165)		59.20% (74)	60.70% (239)	
Ethnicity						
Hispanic/Latino		4.46% (12)		4.80% (6)	4.57% (18)	
Non-Hispanic/Latino		95.54% (257)		95.20% (119)	95.4% (376)	
Education						
Less than high school		29.74% (80)		49.60% (62)	36.04% (142)	
High school		47.96% (129)		38.40% (48)	44.92% (177)	
More than high school		22.30% (60)		12% (15)	19.04% (75)	

Type of offense by recidivism status is reported in Table 4. Overall, the most common charge for current incarceration was a sex crime (29.19%), followed by drug (18.78%) and murder (16.24%) offenses. The overrepresentation of individuals with sex charges is unsurprising given the presence of a sex offender treatment program at one of the prisons used

for data collection.

**Table 4:** Type of offense by recidivism status.

Variable		% (*N*)	
	No Recidivism	Recidivism	Total
Arson	<1% (2)	<1% (1)	<1% (3)
Assault	8.55% (23)	11.2% (14)	9.40% (37)
Crimes Against the State	0% (0)	0% (0)	0% (0)
Drug	17.10% (46)	22.4% (28)	18.78% (74)
Escape	10.41% (28)	12% (15)	10.91% (43)
Fraud	2.60% (7)	1.6% (2)	2.28% (9)
Kidnapping	1.86% (5)	3.2% (4)	2.28% (9)
Misdemeanor	4.1% (11)	14.4% (18)	7.36% (29)
Murder	17.47% (47)	13.6% (17)	16.24% (64)
Negligence	8.92% (24)	12.8% (16)	10.15% (40)
Obstruction of Justice	6.32% (17)	8% (10)	6.85% (27)
Possession of Weapon	8.92% (24)	13.6% (17)	10.41% (41)
Robbery	12.64% (34)	10.4% (13)	11.93% (47)
Sex	37.92% (102)	10.4% (13)	29.19% (115)
Theft	12.64% (34)	12% (15)	12.44% (49)

*Note*. Type of offense reflects the offense of the current incarceration at the time of interview, not of any previous incarcerations. Individuals can have multiple types of offenses.

Lastly, Table 5 reports the presence of mental illness diagnoses as well as symptom count distributions by recidivism status. For the purpose of reporting sample characteristics, diagnoses were grouped into depressive, anxiety, trauma, personality, and substance use disorders. Obsessive compulsive disorder was included in the anxiety category. The rate of SUDs was particularly high in our sample at 86.29% (82.53% for no recidivism and 94.4% for recidivism classes), even when compared to other incarcerated samples.

**Table 5:** Mental health disorders and symptoms by recidivism status.

Variable	% (*N*) or *M (SD)*			
		No Recidivism	Recidivism	Total
ANX		25.65% (69)	24.8% (31)	25.38% (100)
ANX Symptoms		5.32 (6.35)	4.14 (5.98)	4.94 (6.25)
DEP		42% (113)	35.2% (44)	39.84% (157)
DEP Symptoms		3.14 (3.57)	2.88 (3.43)	3.06 (3.53)
PD		55.76% (150)	56% (70)	55.84% (220)
PD Symptoms		16.7 (13.4)	14.4 (13.1)	16.6 (13.3)
PTSD		36.8% (99)	32% (40)	35.28% (139)
PTSD Symptoms		6.03 (5.88)	6.27 (5.98)	6.11 (5.91)
SUD		82.53% (222)	94.4% (118)	86.29% (340)
SUD Symptoms		10.7 (9.66)	13.0 (9.42)	11.4 (9.63)

*Note*. ANX = Anxiety Disorder, DEP = Depressive Disorder, PD = Personality Disorder, PTSD = Posttraumatic Stress Disorder, SUD = Substance Use Disorder. Symptom counts are summed for all disorders within the category. The prevalence of personality disorders was driven by antisocial personality disorder (44.42% of the sample).

### Model comparisons

**Fig 1:**
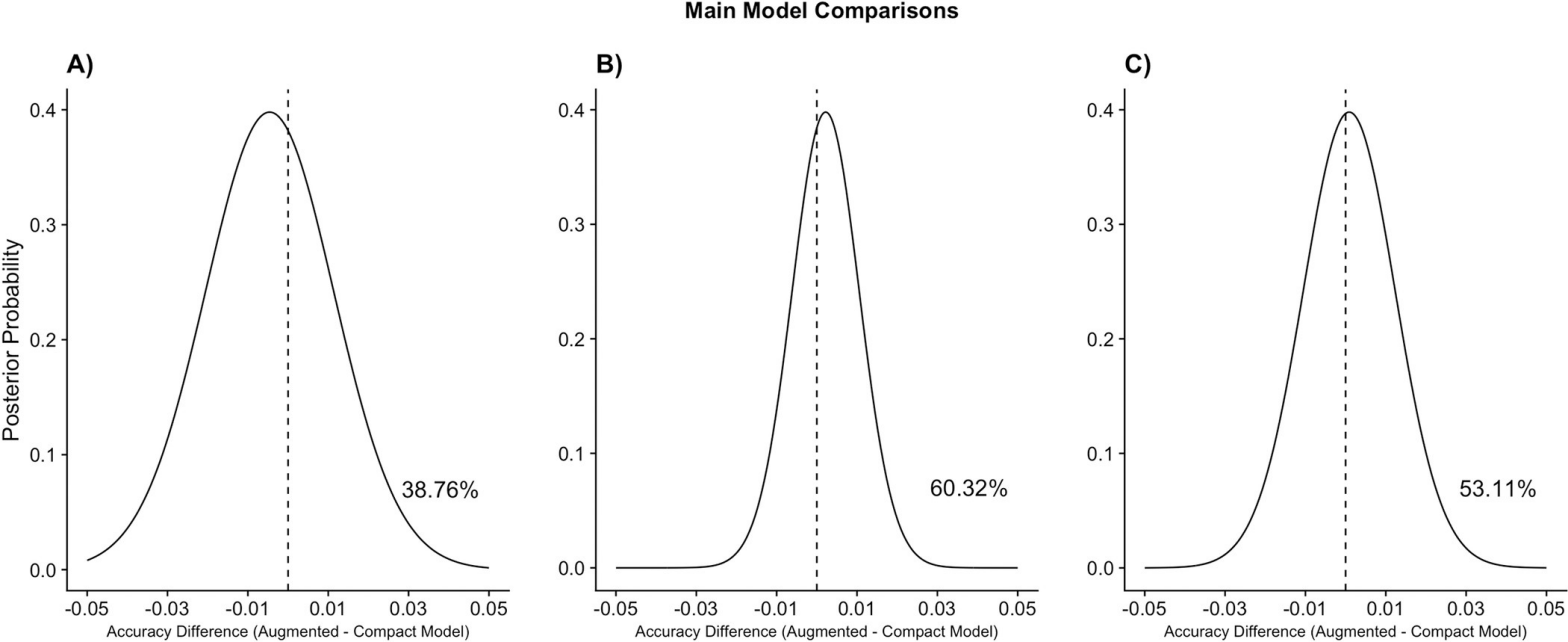
Posterior probabilities for main model comparisons. The vertical dotted line represents equal performance (i.e., no difference in accuracy) between the associated augmented and compact model. A) There is a 38.76% chance that adding both the mental illness and substance use feature sets improves model performance above and beyond the crime and demographic feature sets. B) There is a 60.32% chance that adding the mental illness feature set would improve model performance above and beyond the crime, demographic, and substance use feature sets. C) There is a 53.11% chance that adding the substance use feature set would improve model performance above and beyond the crime, demographic, and mental illness feature sets.

For each model comparison, the posterior probability that the augmented model performed better than the compact model, obtained from the Bayesian correlated t-test, is provided. Greater posterior probabilities (higher percentages) indicate increased likelihood that the focal feature set(s) improve model performance and thus allow us to test the unique contribution that the focal feature set(s) made to recidivism prediction. Posterior probabilities for the main model comparisons are displayed in Fig 1.

As demonstrated in Fig 1A, results showed that there was a 38.76% chance that adding the mental illness and substance use feature sets in the augmented model increased model accuracy above and beyond the crime and demographic feature sets. Fig 1B shows that there was only a 60.32% chance that adding the mental illness feature set would increase model accuracy above and beyond the crime, demographic, and substance use feature sets. Fig 1C shows a similar result: there was a 53.11% chance that adding the substance use feature set would increase model accuracy above and beyond the crime, demographic, and mental illness feature sets. These results indicate that all mental illness and substance use variables as measured in our study did not add incremental predictive value.

### Feature importance

Across the augmented and compact models, four demographic and crime feature categories (sex crime charge, age, education level, and being charged under the age of 18) emerge consistently as the most important feature categories relative to other features in the model. Three of these feature categories (sex crime charge, age, and education level) are noticeably more important than others (i.e., contribute relatively more to predictions). The global importance (mean absolute values of Shapley values) for feature categories for each model configuration (Compact Models 1–3, Augmented Model) appears in S1 Fig.

The combination of age emerging as a globally important feature and our retrospective recidivism definition encouraged us to look more closely at age in our sample. Means and standard deviations of age for individuals overall and by recidivism status appear in Table 3. Additionally, density plots of age by recidivism status appear in S2 Fig.

### Exploratory analyses

Due to the lack of incremental predictive utility from mental illness and substance use variables observed in our model comparisons, we conducted exploratory analyses to investigate if these feature sets had predictive utility when compared to a null model. A null model, also called a no-information model (e.g., an intercept-only linear model), is created by regressing the outcome (recidivism) on no predictors, such that only the mean (proportion) of the outcome variable will influence predictions. These follow-up analyses would clarify whether the mental illness and substance use feature sets did not have any predictive utility or did not add incremental predictive utility over and above the crime and demographics feature sets. We again used Bayesian correlated t-tests to conduct three additional model comparisons: a model with crime and demographics features vs. a null model; a model with mental illness features vs. a null model; and a model with substance use features vs. a null model.

**Fig 2:**
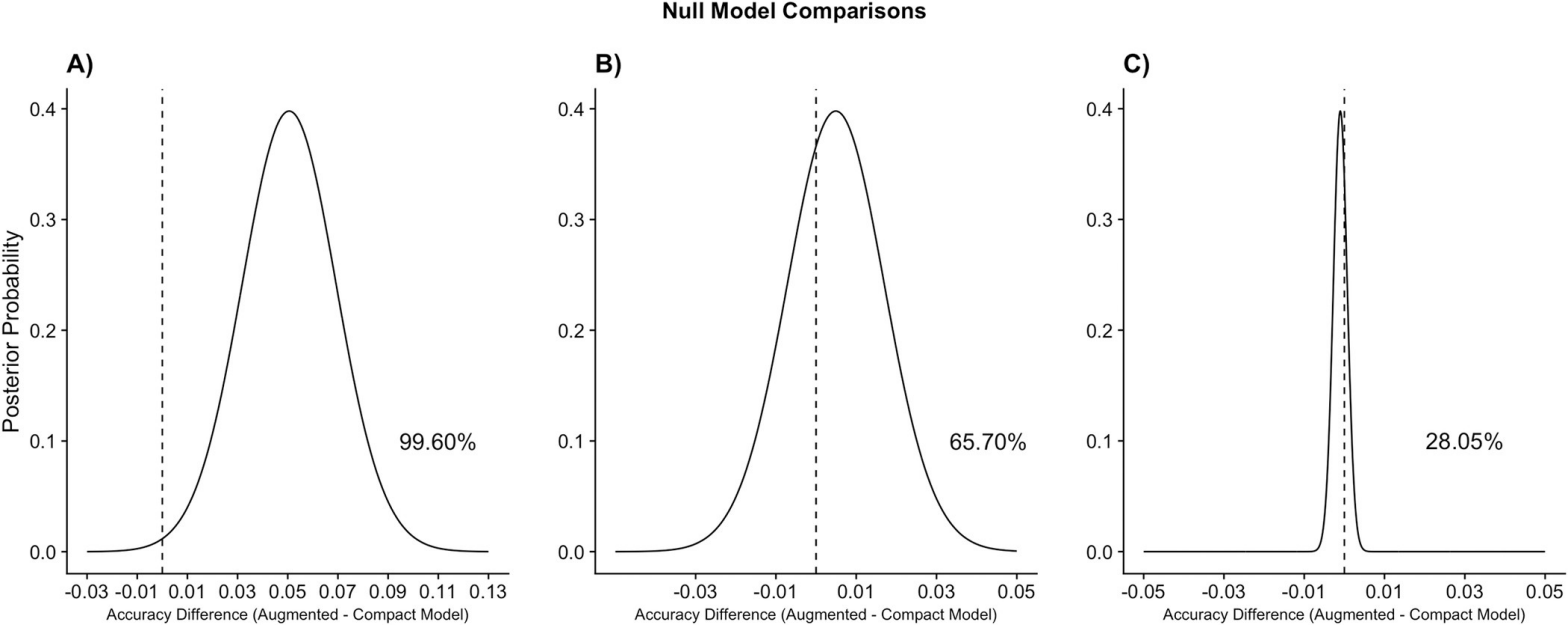
Posterior probabilities for null model comparisons. The vertical dotted line represents equal performance (i.e., no difference in accuracy between the feature set models vs. the null model). A) There is a 99.60% likelihood that a model with crime and demographic variables increases recidivism prediction from a null model. B) There is a 65.70% likelihood that a model with substance use variables increases recidivism prediction from a null model. C) There is a 28.05% likelihood that a model with mental illness variables increases recidivism prediction from a null model.

Posterior probabilities from these comparisons are displayed in Fig 2. There was a 99.60% likelihood that including the crime and demographic feature sets increases model accuracy from a null model, indicating strong signal and clear predictive utility for recidivism (Fig 2A). There was a 65.70% likelihood that the substance use feature set increased model accuracy from a null model (Fig 2B). This result indicates that the substance feature set does not reliably increase predictive utility for recidivism. There was a 28.05% likelihood that including the mental illness feature set increased model accuracy from the null model (Fig 2C). These results indicate that the mental illness feature set did not increase predictive utility for recidivism when compared to a mean-only null model.

## Discussion

This was the first study to use machine learning to understand the relationship between mental illness and recidivism in a general population prison sample in the United States. The results of three model comparisons concluded that mental illness variables and substance use variables did not add incremental predictive utility for recidivism prediction over and above crime and demographic variables. Exploratory analyses comparing the crime and demographic, substance use, and mental illness feature sets to null models found that only the crime and demographics model improved recidivism prediction accuracy. Thus, our findings suggest that in a general population prison sample with no history of bipolar disorder or psychosis, mental illness does not predict recidivism on its own and does not improve recidivism prediction when combined with crime and demographic variables. Our follow-up analyses allowed us to exclude the possibility that the crime and demographics variables were masking the relationship between mental illness and recidivism due to shared variance. If the mental illness and crime and demographics variables shared variance, we would have seen stronger predictive utility of the mental illness and substance use feature sets when compared to a null model. Our results are in line with prior research that has deemphasized the role of mental illness in recidivism prediction [11,42].

The current project builds upon previous work in multiple ways. First, much prior research has focused on forensic psychiatric populations or only individuals with severe mental illness in order to understand the relationship between mental illness and recidivism [9,10,12,14,19,28]. We used a sample that excluded anyone with a history of psychosis or bipolar disorder in order to understand the impacts of other forms of mental illness such as depression, anxiety, PTSD, and personality disorders. Prior research has also largely used administrative data such as intake reports or correctional institution files to characterize mental illness [9,10,12,14,16,43,44]. The current study used a more comprehensive measure of mental illness, the SCID-5, a structured clinical interview that thoroughly assesses for a wide variety of diagnoses and allowed us to obtain both categorical diagnoses and continuous symptom counts. Lastly, we used machine learning, a contemporary statistical method that prioritizes the generalizability of findings via resampling techniques. Resampling increases confidence in our findings as it produces many independent assessments of model performance in data not used for model development (i.e., “new” data). Machine learning also can handle high-dimensional predictor sets, which enabled us to include numerous mental illness variables in our models.

Given the complicated nature of measuring both mental illness and recidivism, we will consider the measurement of these constructs and how they may have impacted our results. We will conclude with a discussion of the ways mental illness impacts involvement in the legal system and how this may be indirectly related to recidivism.

### Recidivism

Our outcome measure was retrospective recidivism, not prospective recidivism—meaning that our models were predicting whether an individual had a prior incarceration in the past, rather than in the future. As a consequence, older individuals were more likely than younger participants to be coded positive for recidivism (Table 3 and S2 Fig). Age emerged as a relatively important feature across all model configurations, among several other demographic and crime features (S1 Fig). This finding supports age as an important feature *relative* to other features in the model. However, because age was included in all models, model comparisons cannot show its unique predictive value. Rather, the model comparisons were considering whether mental illness variables offered unique predictive value above and beyond demographic variables such as age. Age has also emerged as an important feature in research predicting prospective recidivism [8,45].

In addition, while prospective studies have the advantage of prediction of future events, they, too, are limited in their scope. Typical prospective recidivism studies will use a specified time frame to recidivism (e.g., 1–3 years) as the outcome measure. This strategy does not capture those who recidivate after the specified time frame. Although our retrospective study was less likely to categorize younger individuals as recidivists, and therefore there are likely some individuals who were coded as negative for recidivism who will go on to recidivate, it was able to capture a longer time period of recidivism. The current study follows prior research that has utilized retrospective data as a criminal justice outcome [28,35]. Greenberg & Rosenheck (2014) found that a substance use diagnosis was a strong risk factor for a previous incarceration. Using machine learning models in an incarcerated population with schizophrenia, Kirchebner et al. (2020) found that illegal opioid use and anxiety upon discharge were relevant predictors of criminal recidivism; therefore, a signal from mental illness and substance use have been found using a retrospective outcome.

There are opponents to the use of recidivism as a measure of “success” after release from incarceration [46]. Relying on recidivism may lead to incomplete conclusions due to the multitude of ways that personal factors (e.g., race, ethnicity, gender, etc.) intertwine with social factors (e.g., employment opportunities, neighborhood context, etc.) and lead to return to prison or jail. So, it is possible that the effect of mental illness on recidivism is far outweighed by factors like employment and policing, for example, both of which are associated with recidivism [47,48]. Employment rates after release from prison are dismal. Only 55% of individuals reported any earnings in the first year post release, and only 20% of those people earned more than $15,000 [49]. Formerly incarcerated individuals face stigma and background checks that deem them ineligible for many types of employment. Unemployment and an inability to provide for oneself or one’s family, as well as an inability to pay necessary legal fees such as parole fees, may then impact mental illness which can in turn add barriers to finding and maintaining a job with a steady income [50]. In addition, disproportionate policing in communities of color, including higher rates of stop-and-frisk for Black and Hispanic/Latino men in particular, may outweigh the risk for recidivism more than any personal characteristics, such as mental illness [51,52]. The variables in the demographic feature set in the current study only took into account personal attributes (see Table 1), but not the context in which the individual was immersed after release.

In sum, recidivism as an outcome variable is inherently limited. Although it is a useful indicator of one type of success after release from incarceration, it may be too simple to measure without larger social context. We included personal identity factors such as age, race, ethnicity, and gender in our models, as well as both personal and parental education levels which have been used as a proxy for socio-economic status [53]. However, we were not able to account for social factors such as employment opportunities and neighborhood context that may impact recidivism directly or indirectly–including by impacting mental illness.

### Substance use

It was surprising that the substance use feature set did not increase predictive utility, as the relationship between substance use and recidivism is widely accepted in the literature [17,18]. However, an overwhelming majority of our sample met criteria for a history of SUD (86%). The lack of variability therefore may have hidden the impact of substance use. Previous studies that have found a clear relationship between substance use disorders and recidivism have had more balance with respect to SUD diagnoses among their participants. For example, 52% of subjects in Wilson and Wood (2014), 59% in Sadeh and McNeil (2015), and 68% in Walters & Crawford (2013). Each of these studies utilized DOC files in order to obtain SUD diagnoses, which likely captured only the most severe cases of SUD. A strength of the current project was the use of the SCID-5, which provided a more comprehensive assessment of mental illness including identifying individuals with mild or moderate SUD diagnoses. The contrast between the lack of predictive utility in our study compared to positive findings in other research that has relied on DOC files for diagnosis, then, may suggest that only severe SUD at the time of arrest can predict recidivism.

Additionally, our SCID-5 data did not provide us with a temporally specific estimate of substance use or other mental illness disorders. For example, a participant who had been in remission from an opioid use disorder for 10 years prior to their incarceration would be coded the same (“yes”) as someone who had an opioid use disorder at the time of arrest. One third of people in prison report that they were under the influence of drugs or alcohol at the time of their offense [54]. It is possible that with more temporal specificity, we would have replicated the additive value of substance use features in the prediction of recidivism.

### Mental illness beyond substance use

The experience of mental illness symptomatology across diagnoses is dynamic. Static diagnostic measurement may not capture the precision needed to investigate complex relationships, like the one between recidivism and mental illness. Some researchers have utilized ecological momentary assessment (EMA), a methodological approach that involves repeated sampling of symptoms throughout the day, to provide a more in-depth picture of the fluctuations in symptom severity [55,56]. EMA has shown to be more effective than self-report measures for demonstrating clinically meaningful change [56]. In this study, we were interested in whether a more comprehensive measurement of history of mental illness would increase predictive accuracy of recidivism. However, research on the relationship between mental illness and recidivism could benefit from a more temporally precise estimate of mental illness symptomatology during the period of release between prison sentences. A more temporally precise estimate of mental illness could also include a record of mental health treatment utilization. Treatment can significantly alter the course and outcome of both mental health disorders and pre- and post-release adjustment.

Lastly, although we were able to investigate the relationship between mental illness and recidivism in a general population sample without history of psychosis or bipolar disorder, it is possible that we would have found more predictive signal from the mental illness data if individuals with these severe mental illnesses were included. Prior research demonstrating a relationship between mental illness and criminal justice outcomes such as recidivism for a new crime or reincarceration after a parole violation has typically utilized samples with more severe forms of mental illness including psychotic disorders [12,15,16].

### Mental illness and the legal system

Although the current study does not support the idea that mental illness, excluding psychosis and bipolar disorder, increases risk for recidivism, it is imperative to understand why mental health treatment during incarceration is necessary and how it could have an indirect impact on recidivism. First, the rates of mental illness in our sample were high (Table 5), highlighting there is critical need for treatment in this population. Also, there are critical human rights, safety, and rehabilitation concerns for incarcerated people with mental illness. Withholding adequate medical and mental healthcare from incarcerated individuals was ruled cruel and unusual punishment by the U.S. Supreme Court [57,58]. *Ruiz v*. Estelle (1980) mandated that prisons implement a systemic program for screening and evaluating mental health, required that treatment consist of more than transfer to segregated housing, and necessitated a sufficient number of trained mental health professionals on staff to treat and identify mental illness. Although research shows that many institutions offer mental healthcare on a superficial basis [59], there remains a legal imperative to provide mental health care to incarcerated individuals who need it. Engagement in treatment with a compassionate clinician is one way that incarcerated individuals can begin to rebuild a sense of personal dignity, which can lead to desistance from crime once released [60].

Mental illness can also disrupt aspects of the correctional environment. For example, individuals with mental illness receive more disciplinary infractions than those without mental illness [61–64]. Individuals with major depression as well as psychosis are more likely to receive all types of infractions, including those that involve aggression [62]. Those with co-occurring mental illness and substance use at the time of admission report more variety and number of infractions when compared to those without co-occurring psychiatric disorders [63]. Infractions can lead to time in solitary confinement, and placement in solitary confinement increases the odds of both general and violent recidivism [65].

Mental illnesses such as depression and anxiety may also interfere with the ability to engage in rehabilitation programs that are aimed at reducing recidivism such as anger management, substance use treatment, and vocational training. There is evidence that treatment of PTSD with cognitive behavioral therapy (CBT) is associated with a decrease in anger [66] and that using CBT for the treatment of depression in those with SUD decreases not only depressive symptoms but also substance use symptoms [67]. Therefore, treating mental illness could increase positive outcomes for rehabilitation programs. Given the evidence that engagement in vocational training during incarceration reduces recidivism and the evidence that unemployment contributes to recidivism, treating mental illness could also be a vital step to improving employment outcomes [49,68]

## Conclusion

Using rigorous machine learning methods that prioritize prediction in new data, the current study found that mental illness and substance use variables did not add unique predictive utility of recidivism over and above crime and demographic variables. We propose that the higher rates of SUDs in our sample compared to other incarcerated samples may have hidden the impact of substance use as a predictor. We also discuss larger structural and systemic issues, such as difficulty finding employment after release from prison and higher rates of policing in communities of color, which may supersede the predictive value of individual characteristics like mental illness diagnoses and symptoms. Despite not finding a direct relationship between mental illness and recidivism, treatment of mental illness in incarcerated populations is still essential due to the high rates of mental illness, the legal imperative, the possibility of decreasing institutional disciplinary burden, the opportunity to increase the effectiveness of rehabilitation programs in prison, and the potential to improve meaningful outcomes beyond recidivism following release.

## Supporting information

S1 Text Transparency checklist. (DOC)

S1 Fig Feature importance. The global importance (mean absolute values of Shapley values) for feature categories for each model configuration (Compact Models 1–3, Augmented Model).
(TIF)

S2 Fig Age density plot by recidivism status. (TIF)

S1 Dataset (CSV)

## References

[1] P. Wagner and W. Sawyer, “States of Incarceration: The Global Context 2018 |Prison Policy Initiative.” Accessed: Apr. 04, 2020. [Online]. Available: https://www.prisonpolicy.org/global/2018.html

[2] Prins S. J., “Prevalence of mental illnesses in U.S. state prisons: A systematic review,” *Psychiatr*. *Serv*., 2014, doi: 10.1176/appi.ps.201300166 24686574 PMC4182175

[3] NAMI, “Jailing People with Mental Illness | NAMI: National Alliance on Mental Illness.” Accessed: Apr. 04, 2020. [Online]. Available: https://www.nami.org/Learn-More/Mental-Health-Public-Policy/Jailing-People-with-Mental-Illness

[4] James D. J. and Glaze L. E., “Bureau of Justice Statistics Special Report Mental Health Problems of Prison and Jail Inmates,” *Bur*. *Justice Stat*. *Spec*. *Rep*., 2006.

[5] Sawyer W., “Since you asked: How many people are released from each state’s prisons and jails every year?,” Prison Policy Initiative. Accessed: Nov. 21, 2022. [Online]. Available: https://www.prisonpolicy.org/blog/2022/08/25/releasesbystate/

[6] Alper M., Durose M. R., Statisticians B., Markman J., and former B. Statisticians, “Special Report 2018 Update on Prisoner Recidivism: A 9-Year Follow-up Period (2005–2014),” 2018.

[7] Burgess E. W., “Factors determining success or failure on parole.,” in *The workings of the indeterminate sentence law and the parole system in Illinois*, 1928.

[8] Katsiyannis A., Whitford D. K., Zhang D., and Gage N. A., “Adult Recidivism in United States: A Meta-Analysis 1994–2015,” *J*. *Child Fam*. *Stud*., 2018, doi: 10.1007/s10826-017-0945-8

[9] Gagliardi G. J., Lovell D., Peterson P. D., and Jemelka R., “Forecasting recidivism in mentally ill offenders released from prison,” *Law Hum*. *Behav*., 2004, doi: 10.1023/b:lahu.0000022319.03637.45 15141775

[10] Hall D. L., Miraglia R. P., Lee L. W. G., Chard-Wierschem D., and Sawyer D., “Predictors of general and violent recidivism among SMI prisoners returning to communities in new york state,” *J*. *Am*. *Acad*. *Psychiatry Law*, 2012. 22635294

[11] Bonta J., Blais J., and Wilson H. A., “A theoretically informed meta-analysis of the risk for general and violent recidivism for mentally disordered offenders,” *Aggression and Violent Behavior*. 2014. doi: 10.1016/j.avb.2014.04.014

[12] Cloyes K. G., Wong B., Latimer S., and Abarca J., “Time to prison return for offenders with serious mental illness released from prison: A survival analysis,” *Crim*. *Justice Behav*., 2010, doi: 10.1177/0093854809354370

[13] Igoumenou A., Kallis C., and Coid J., “Treatment of psychosis in prisons and violent recidivism,” *BJPsych Open*, 2015, doi: 10.1192/bjpo.bp.115.000257 27703740 PMC4995573

[14] King E. A., Tripodi S. J., and Veeh C. A., “The Relationship Between Severe Mental Disorders and Recidivism in a Sample of Women Released from Prison,” *Psychiatr*. *Q*., vol. 89, no. 3, pp. 717–731, Sep. 2018, doi: 10.1007/s11126-018-9572-9 29520740

[15] Louden J. E. and Skeem J. L., “Parolees with Mental Disorder: Toward Evidence-Based Practice,” *US Irvine Cent*. *Evid*. *Based Correct*.*—Bull*., 2011.

[16] Sadeh N. and McNiel D. E., “Posttraumatic Stress Disorder Increases Risk of Criminal Recidivism Among Justice-Involved Persons With Mental Disorders,” *Crim*. *Justice Behav*., 2015, doi: 10.1177/0093854814556880

[17] Wilson J. A. and Wood P. B., “Dissecting the relationship between mental illness and return to incarceration,” *J*. *Crim*. *Justice*, 2014, doi: 10.1016/j.jcrimjus.2014.09.005

[18] Dowden C. and Brown S. L., “The role of substance abuse factors in predicting recidivism: A meta-analysis,” *Psychol*. *Crime Law*, 2002, doi: 10.1080/10683160208401818

[19] Pflueger M. O., Franke I., Graf M., and Hachtel H., “Predicting general criminal recidivism in mentally disordered offenders using a random forest approach,” *BMC Psychiatry*, 2015, doi: 10.1186/s12888-015-0447-4 25885691 PMC4384374

[20] Haarsma G., Davenport S., White D. C., Ormachea P. A., Sheena E., and Eagleman D. M., “Assessing Risk Among Correctional Community Probation Populations: Predicting Reoffense With Mobile Neurocognitive Assessment Software,” *Front*. *Psychol*., 2020, doi: 10.3389/fpsyg.2019.02926 32038355 PMC6992536

[21] Garb H. N. and Wood J. M., “Methodological advances in statistical prediction,” *Psychol*. *Assess*., 2019, doi: 10.1037/pas0000673 30855159

[22] Duwe G. and Kim K. D., “Out With the Old and in With the New? An Empirical Comparison of Supervised Learning Algorithms to Predict Recidivism,” *Crim*. *Justice Policy Rev*., 2017, doi: 10.1177/0887403415604899

[23] Zeng J., Ustun B., and Rudin C., “Interpretable classification models for recidivism prediction,” *J*. *R*. *Stat*. *Soc*. *Ser*. *A Stat*. *Soc*., 2017, doi: 10.1111/rssa.12227

[24] Wang P., Mathieu R., Ke J., and Cai H. J., “Predicting criminal recidivism with support vector machine,” in *2010 International Conference on Management and Service Science*, *MASS* 2010, 2010. doi: 10.1109/ICMSS.2010.5575352

[25] Silver E. and Chow-Martin L., “A multiple models approach to assessing recidivism risk: Implications for judicial decision making,” *Crim*. *Justice Behav*., 2002, doi: 10.1177/009385402236732

[26] Liu Y. Y., Yang M., Ramsay M., Li X. S., and Coid J. W., “A Comparison of Logistic Regression, Classification and Regression Tree, and Neural Networks Models in Predicting Violent Re-Offending,” *J*. *Quant*. *Criminol*., 2011, doi: 10.1007/s10940-011-9137-7

[27] Constantine R. J. et al., “Arrest trajectories of adult offenders with a serious mental illness,” *Psychol*. *Public Policy Law*, 2010, doi: 10.1037/a0020852

[28] Kirchebner J., Philipp Günther M., and Lau S., “Identifying influential factors distinguishing recidivists among offender patients with a diagnosis of schizophrenia via machine learning algorithms,” *Forensic Sci*. *Int*., 2020, doi: 10.1016/j.forsciint.2020.110435 32784039

[29] F. Schönbrodt, M. Heene, and L.-M.-U. München, “The Replication-/Credibility-Crisis in Psychology: Consequences at LMU?”.

[30] Aczel B. et al., “A consensus-based transparency checklist,” *Nat*. *Hum*. *Behav*., vol. 4, no. 1, Art. no. 1, Jan. 2020, doi: 10.1038/s41562-019-0772-6 31792401 PMC8324470

[31] D. Wechsler, *Manual for the Wechsler Adult Intelligence Scale—Revised*. 1981. Thesis_references-Converted #317.

[32] Hare R. D., *The Hare Psychopathy Checklist—Revised*. 1991.

[33] First M. B. and Gibbon M., *The Structured Clinical Interview for DSM-IV Axis I Disorders (SCID-I) and the Structured Clinical Interview for DSM-IV Axis II Disorders (SCID-II)*. 2004. doi: 10.1002/9780471726753

[34] First M. B., Williams J. B. W., Karg R. S., and Spitzer R. L., “Structured clinical interview for DSM-5, Research Version.,” *Am*. *Psychiatr*. *Assoc*. *Wash*. *DC*, 2015.

[35] Greenberg G. A. and Rosenheck R. A., “Psychiatric correlates of past incarceration in the national co-morbidity study replication,” *Crim*. *Behav*. *Ment*. *Health*, vol. 24, no. 1, pp. 18–35, Feb. 2014, doi: 10.1002/cbm.1875 23881907

[36] Wickham H. et al., “Welcome to the Tidyverse,” *J*. *Open Source Softw*., vol. 4, no. 43, p. 1686, Nov. 2019, doi: 10.21105/joss.01686

[37] Kuhn M. and Johnson K., *Applied Predictive Modeling*. New York, NY: Springer, 2013.

[38] Benavoli A., Corani G., Demšar J., and Zaffalon M., “Time for a change: a tutorial for comparing multiple classifiers through Bayesian analysis,” *J*. *Mach*. *Learn*. *Res*., vol. 18, no. 1, pp. 2653–2688, 2017.

[39] Lundberg S. M. and Lee S.-I., “A Unified Approach to Interpreting Model Predictions,” in *Advances in Neural Information Processing Systems*, Curran Associates, Inc., 2017. Accessed: Oct. 16, 2023. [Online]. Available: https://proceedings.neurips.cc/paper_files/paper/2017/hash/8a20a8621978632d76c43dfd28b67767-Abstract.html

[40] WI DOC, “Recidivism After Release From Prison.” Accessed: May 27, 2021. [Online]. Available: https://doc.wi.gov/DataResearch/InteractiveDashboards/RecidivismAfterReleaseFromPrison_2.pdf

[41] WI DOC, “DOC DAI—Admissions to Prison Dashboard.” Accessed: May 27, 2021. [Online]. Available: https://doc.wi.gov/Pages/DataResearch/PrisonAdmissions.aspx

[42] Skeem J. L., Manchak S., and Peterson J. K., “Correctional policy for offenders with mental illness: Creating a new paradigm for recidivism reduction,” *Law Hum*. *Behav*., 2011, doi: 10.1007/s10979-010-9223-7 20390443

[43] Fisher W. H., Hartwell S. W., Deng X., Pinals D. A., Fulwiler C., and Roy-Bujnowski K., “Recidivism Among Released State Prison Inmates Who Received Mental Health Treatment While Incarcerated,” *Crime Delinquency*, vol. 60, no. 6, pp. 811–832, 2014, doi: 10.1177/0011128714541204

[44] Hartwell S., Fisher W., Deng X., Pinals D. A., and Siegfriedt J., “Intensity of offending following state prison release among persons treated for mental health problems while incarcerated,” *Psychiatr*. *Serv*., vol. 67, no. 1, pp. 49–54, Jan. 2016, doi: 10.1176/appi.ps.201400417 26278228

[45] Gendreau P., Little T., and Goggin C., “A meta-analysis of the predictors of adult offender recidivism: What Works!,” *Criminology*, 1996, doi: 10.1111/j.1745-9125.1996.tb01220.x

[46] National Academies of Sciences Engineering and Medicine, *The Limits of Recidivism*: *Measuring Success After Prison*. Washington, DC: The National Academies Press, 2022. doi: 10.17226/26459

[47] J. A. Butts and V. Schiraldi, “The Recidivism Trap,” The Marshall Project. Accessed: May 07, 2021. [Online]. Available: https://www.themarshallproject.org/2018/03/14/the-recidivism-trap

[48] Harer M. D., “Recidivism among federal prisoners released in 1987,” *J*. *Correct*. *Educ*., vol. 46, no. 3, pp. 98–128, 1995.

[49] A. Looney and N. Turner, “Work and opportunity before and after incarceration.” Mar. 2018. [Online]. Available: https://www.brookings.edu/wp-content/uploads/2018/03/es_20180314_looneyincarceration_final.pdf

[50] Paul K. I. and Moser K., “Unemployment impairs mental health: Meta-analyses,” *J*. *Vocat*. *Behav*., vol. 74, no. 3, pp. 264–282, Jun. 2009, doi: 10.1016/j.jvb.2009.01.001

[51] T. Lau, “Predictive Policing Explained.” Accessed: May 01, 2021. [Online]. Available: https://www.brennancenter.org/our-work/research-reports/predictive-policing-explained

[52] NYCLU, “Stop-and-Frisk in the De Blasio Era,” New York Civil Liberties Union. Accessed: May 01, 2021. [Online]. Available: https://www.nyclu.org/en/stop-and-frisk-data

[53] Hollingshead A. B., “Four factor index of social status. New Haven,” *CT Yale Univ*., 1975.

[54] Bureau of Justice Statistics, “Drug Use and Crime.” Accessed: Nov. 23, 2022. [Online]. Available: https://bjs.ojp.gov/drugs-and-crime-facts/drug-use-and-crime

[55] Firth J., Torous J., and Yung A. R., “Ecological momentary assessment and beyond: The rising interest in e-mental health research,” *J*. *Psychiatr*. *Res*., vol. 80, pp. 3–4, Sep. 2016, doi: 10.1016/j.jpsychires.2016.05.002 27236099

[56] Moore R. C., Depp C. A., Wetherell J. L., and Lenze E. J., “Ecological momentary assessment versus standard assessment instruments for measuring mindfulness, depressed mood, and anxiety among older adults,” *J*. *Psychiatr*. *Res*., vol. 75, pp. 116–123, Apr. 2016, doi: 10.1016/j.jpsychires.2016.01.011 26851494 PMC4769895

[57] “Estelle v. Gamble,” Justia Law. Accessed: May 03, 2021. [Online]. Available: https://supreme.justia.com/cases/federal/us/429/97/

[58] “Ruiz v. Estelle,” Justia Law. Accessed: May 03, 2021. [Online]. Available: https://law.justia.com/cases/federal/district-courts/FSupp/503/1265/1466998/

[59] Cohen T. R., Mujica C. A., Gardner M. E., Hwang M., and Karmacharya R., “Mental Health Units in Correctional Facilities in the United States,” *Harv*. *Rev*. *Psychiatry*, vol. 28, no. 4, pp. 255–270, Jul. 2020, doi: 10.1097/HRP.0000000000000267 32692089

[60] Fantuzzo J. P., “Recognizing human dignity behind bars: A moral justification for college-in-prison programs,” *Theory Res*. *Educ*., vol. 20, no. 1, pp. 26–43, Mar. 2022, doi: 10.1177/14778785221102035

[61] Walters G. D. and Crawford G., “In and out of prison: Do importation factors predict all forms of misconduct or just the more serious ones?,” *J*. *Crim*. *Justice*, vol. 41, no. 6, pp. 407–413, Nov. 2013, doi: 10.1016/j.jcrimjus.2013.08.001

[62] Felson R. B., Silver E., and Remster B., “Mental Disorder and Offending in Prison,” *Crim*. *Justice Behav*., vol. 39, no. 2, pp. 125–143, Feb. 2012, doi: 10.1177/0093854811428565

[63] Wood S. R., “State prisoner misconduct: Contribution of dual psychiatric and substance use disorders,” *Int*. *J*. *Forensic Ment*. *Health*, vol. 13, no. 4, pp. 279–294, Oct. 2014, doi: 10.1080/14999013.2014.951108

[64] Wood S. R., “Co-occurring serious mental illnesses and substance use disorders as predictors of assaultive infraction charges among adult male jail inmates,” *J*. *Forensic Psychiatry Psychol*., vol. 29, no. 2, pp. 189–210, Mar. 2018, doi: 10.1080/14789949.2017.1352015

[65] Luigi M., Dellazizzo L., Giguère C.-É., Goulet M.-H., Potvin S., and Dumais A., “Solitary Confinement of Inmates Associated With Relapse Into Any Recidivism Including Violent Crime: A Systematic Review and Meta-Analysis,” *Trauma Violence Abuse*, vol. 23, no. 2, pp. 444–456, Apr. 2022, doi: 10.1177/1524838020957983 32935639

[66] Cahill S. P., Rauch S. A., Hembree E. A., and Foa E. B., “Effect of Cognitive-Behavioral Treatments for PTSD on Anger,” *J*. *Cogn*. *Psychother*., vol. 17, no. 2, pp. 113–131, Apr. 2003, doi: 10.1891/jcop.17.2.113.57434

[67] Watkins K. E. et al., “An Effectiveness Trial of Group Cognitive Behavioral Therapy for Patients With Persistent Depressive Symptoms in Substance Abuse Treatment,” *Arch*. *Gen*. *Psychiatry*, vol. 68, no. 6, pp. 577–584, Jun. 2011, doi: 10.1001/archgenpsychiatry.2011.53 21646576 PMC3230556

[68] Mohammed H. and Mohamed W. A. W., “Reducing Recidivism Rates through Vocational Education and Training,” *Procedia—Soc*. *Behav*. *Sci*., vol. 204, pp. 272–276, Aug. 2015, doi: 10.1016/j.sbspro.2015.08.151

