# Supplementary material for: Clarifying the relationship between mental illness and recidivism using machine learning: A retrospective study
Source: PLoS One. 2024 Feb 23;19(2):e0297448. doi: 10.1371/journal.pone.0297448 (PMC10890739; doi:10.1371/journal.pone.0297448)
Supplement: S1 Text — (DOC) [file pone.0297448.s001.doc]

Appendix 1: Transparency Checklist

Using machine learning methods to clarify the relationship between mental illness and recidivism

Transparency Report 1.0 (full, 36 items)

[masked]

23/11/2022

Corresponding author’s email address: [[masked]](jjcurtin@wisc.edu)

Link to Project Repository:

## PREREGISTRATION SECTION

Prior to analyzing the complete data set, a time-stamped preregistration was posted in an independent, third-party registry for the data analysis plan. **No**

**Comments about your Preregistration**

Our research questions remained constant throughout this project, but our analysis plan evolved as we improved our understanding of these analytic methods that remain somewhat novel to our research group. These factors, combined with increasing knowledge of the data with each analysis iteration, made this project inappropriate for preregistration. Critically, our most important analytic decision – to use the Bayesian correlated t-test – was selected at project outset during a master’s thesis proposal and never changed.

## METHODS SECTION

**The manuscript fully describes…**

the rationale for the sample size used (e.g., an a priori power analysis). **Yes**

how participants were recruited. **Yes**

how participants were selected (e.g., eligibility criteria). **Yes**

what compensation was offered for participation. **Yes**

how participant dropout was handled (e.g., replaced, omitted, etc). **NA**

how participants were assigned to conditions. **NA**

how stimulus materials were randomized. **NA**

whether (and, if so, how) participants, experimenters, and data-analysts were kept naive to potentially biasing information. **NA**

the study design, procedures, and materials to allow independent replication. **Yes**

the measures of interest (e.g., friendliness). **Yes**

all operationalizations for the measures of interest (e.g., a questionnaire measuring friendliness). **Yes**

**Comments about your Methods section**

No comments.

## RESULTS AND DISCUSSION SECTION

**The manuscript…**

distinguishes explicitly between “confirmatory” (i.e., prespecified) and “exploratory” (i.e., not prespecified) analyses. **Yes**

describes how violations of statistical assumptions were handled. **Yes**

justifies all statistical choices (e.g., including or excluding covariates; applying or not applying transformations; use of multi-level models vs. ANOVA). **Yes**

reports the sample size for each cell of the design. **NA**

reports how incomplete or missing data were handled. **Yes**

presents protocols for data preprocessing (e.g., cleaning, discarding of cases and items, normalizing, smoothing, artifact correction). **Yes**

**Comments about your Results and Discussion**

No comments.

## DATA, CODE, AND MATERIALS AVAILABILITY SECTION

**The following have been made publicly available…**

the (processed) data, on which the analyses of the manuscript were based. **No**

all code and software (that is not copyright protected). **No**

all instructions, stimuli, and test materials (that are not copyright protected). **No**

The manuscript includes a statement concerning the availability and location of all research items, including data, materials, and code relevant to the study. **NA**

**Comments about your Data, Code, and Materials**

Data are unable to be shared publicly given the sensitivity of data from incarcerated individuals.

## References

Aczel, B., Szaszi, B., Sarafoglou, A. Kekecs, Z., Kucharský, Š., Benjamin, D., … & Wagenmakers, E.-J. (2019). A consensus-based transparency checklist. *Nature Human Behaviour*, 1–3. <doi:10.1038/s41562-019-0772-6>

**Appendix 2: Supplemental Figures**

**Fig S1. Global Shapley values across models.** Global importance (mean absolute value of Shapley value) is displayed for feature categories for each model (Compact Models 1-3, Augmented Model). Values are shown for the 15 most important feature categories for each model, ordered by their aggregate global importance (i.e., total bar length). Longer bars indicate greater relative global importance. Feature categories are colored by feature set (i.e., Crime & Demographic features, Substance Use features, and Mental Illness features).

**Fig S2. Density plot of age by recidivism status.** Density plots of age by recidivism status show the distributions of age for participants who have or have not recidivated.
