# Supplementary figures and images for: Clarifying the relationship between mental illness and recidivism using machine learning: A retrospective study
Source: PLoS One. 2024 Feb 23;19(2):e0297448. doi: 10.1371/journal.pone.0297448 (PMC10890739; doi:10.1371/journal.pone.0297448)

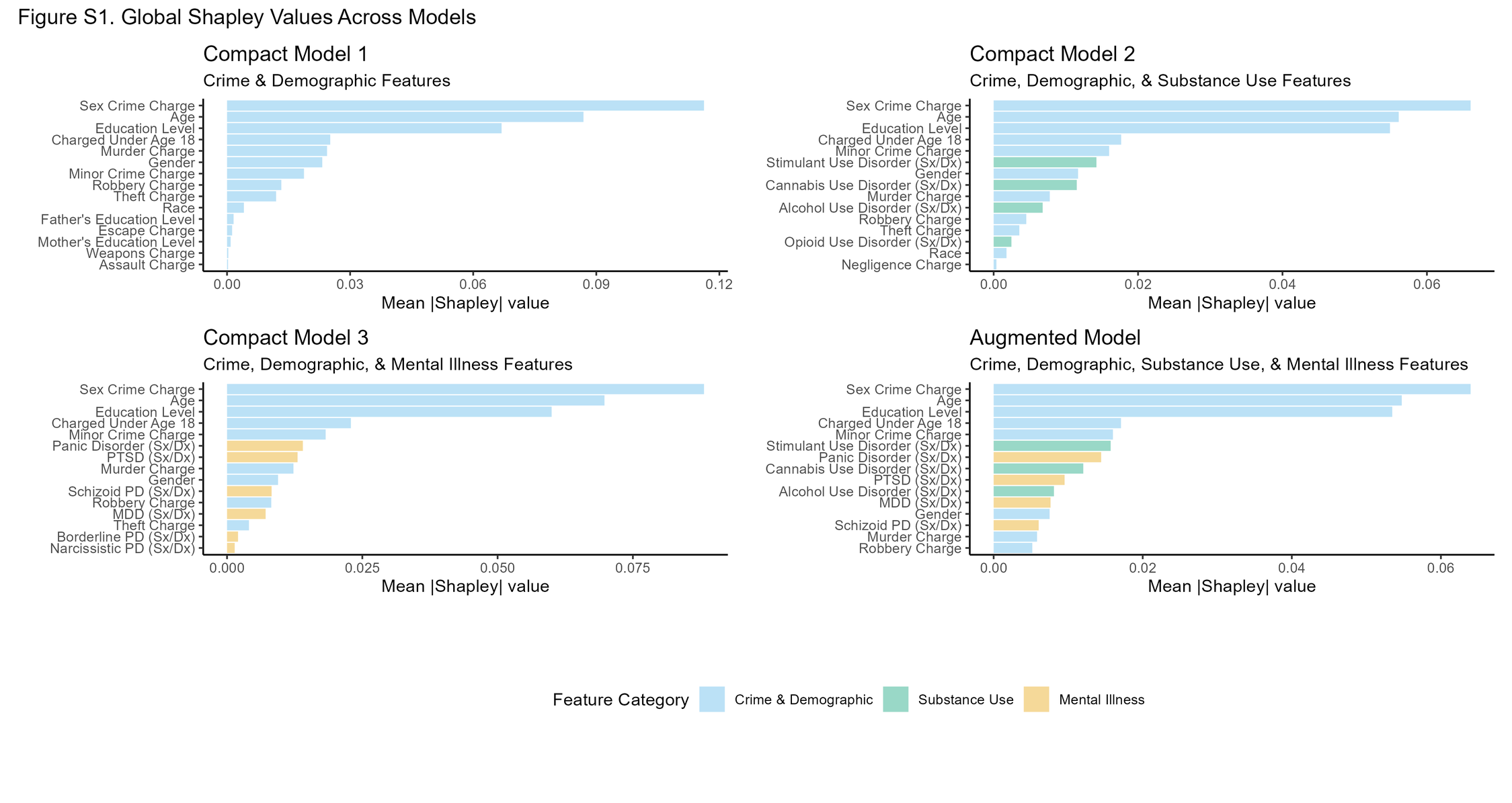

Supplement: S1 Fig — The global importance (mean absolute values of Shapley values) for feature categories for each model configuration (Compact Models 1–3, Augmented Model). (TIF) [file pone.0297448.s002.tif]

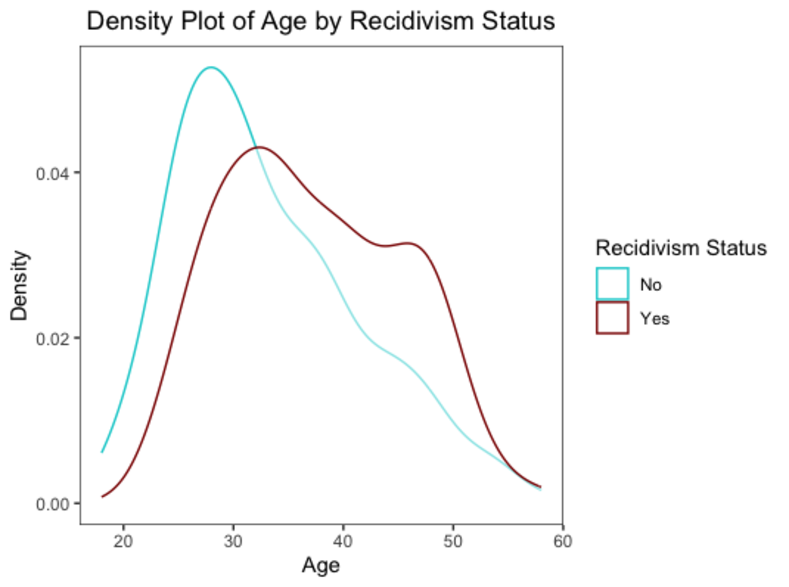

Supplement: S2 Fig — (TIF) [file pone.0297448.s003.tif]
